# *In vitro* effect of leptin on human cardiac contractility

**DOI:** 10.1017/jns.2019.6

**Published:** 2019-04-10

**Authors:** Ryan Chaban, Katja Buschmann, Ahmed Ghazy, Alicia Poplawski, Nadja Wittmann, Andres Beiras-Fernandez, Christian-Friedrich Vahl

**Affiliations:** 1 Department of Cardiothoracic and Vascular Surgery, University Medical Center of the Johannes Gutenberg University Mainz, Langenbeckstr. 1, 55131 Mainz, Germany; 2 Institute of Medical Biostatistics, Epidemiology and Informatics (IMBEI), University Medical Center of the Johannes Gutenberg University Mainz, Langenbeckstr. 1, 55131 Mainz, Germany; 3 Johannes Gutenberg University Mainz, 55122 Mainz, Germany

**Keywords:** Obesity, Leptin, Contraction force, Cardiac contractility, CD, contraction duration, CF, maximal isometric contraction force, KH, Krebs–Henseleit

## Abstract

Leptin, a hormone produced by adipose tissue, has been linked to many regulatory pathways. Its role in the complex relationship between obesity and CVD is not yet clear. The aim of the present study was to evaluate whether leptin interferes directly with cardiac function regulation, altering its contractile force character, and hence contributing to different pathological processes. Muscle samples were obtained from human atrial myocardium. Each trial included two samples from the same patient. They were simultaneously electrically stimulated under sustained perfusion to perform isometric contractions. One sample was treated with a high concentration of human recombinant leptin (1 µg/ml). The other was treated with placebo and served as a control. The exhibited contraction forces (CF) and the contraction duration (CD) after 20 min of treatment were normalised by dividing them by the values before the treatment and reported as a percentage. A total of ten successful trials were conducted. Exposure to leptin did not yield a statistically significant variation in both CF and CF. In the treatment group, CF% measured 108 (95 % CI 91, 125) % and CD% measured 95 (95 % CI 90, 101) % after 20 min. In the control group, CF% measured 105 (90 % CI 84, 126) % and CD% measured 92 (95 % CI 80, 105) % after 20 min. We concluded that leptin does not alter the contractile character of human atrial tissues, even in supraphysiological dosage. These results suggest that leptin does not play a role in short-term cardiac regulation.

Over the last four decades, the prevalence of obesity has been growing continually, afflicting more people than ever^(^^1^^,^^2^^)^. According to the WHO, obesity has nearly tripled worldwide since 1975, with almost 39 % of adults being overweight (BMI greater than or equal to 25 kg/m^2^) and 13 % being obese (BMI greater than or equal to 30 kg/m^2^) in 2016^(^^3^^)^. At least 2·8 million people die each year as a result of being overweight or obese, according the Global Health Observatory^(^^4^^)^, which also states that overweight and obesity correlate with adverse metabolic effects on blood pressure, cholesterol, TAG and insulin resistance, increased risks of CHD, ischaemic stroke and type 2 diabetes mellitus and higher incidence of cancer of breast, colon, prostate, endometrium, kidney and gall bladder^(^^4^^)^.

The relationship between obesity and CVD is one of the most studied and complex medical topics due to a wide range of pro-inflammatory and pro-atherosclerotic mechanisms^(^^5^^)^ and it seems there is a consent in the medical world about the cumulative negative effect of obesity on the cardiovascular system^(^^6^^,^^7^^)^. Obesity has been reported to increase the risk of atrial fibrillation^(^^8^^)^ and be an independent risk factor for heart failure^(^^9^^,^^10^^)^ and both all-cause and cardiovascular mortality^(^^11^^)^. It is worth mentioning here that some studies describe higher survival rates in critical ill obese patients against critical ill individuals of normal weight, giving this hard-to-explain paradox the name ‘obesity paradox’^(^^12^^,^^13^^)^.

In a previous study of ours, we were able to verify the correlation between BMI and reduced cardiac function *in vitro*. Using cardiac atrial tissues, obtained from patients undergoing cardiac surgery, we found that active contraction forces and passive resting tension decline significantly with increasing body weight. The ratio between passive resting tension/active contraction forces correlated significantly with body weight. The negative association between body weight and active tension amplitude was more pronounced in women^(^^14^^)^. This correlation was also reported by Broussard *et al*.^(^^15^^)^, who found in an experimental model that high-fat feeding canines significantly increased body weight and reduced left ventricular function.

The role of leptin, the ‘appetite’ hormone produced by fat tissues, in the relationship between obesity and CVD has been suggested in many studies^(^^16^^–^^18^^)^, as it is usually found in higher levels in obese individuals, signifying the presence of so-called leptin resistance^(^^19^^,^^20^^)^. Leptin has been linked to pro-inflammatory activity in other studies^(^^21^^–^^23^^)^ and to a direct effect on cardiomyocyte contraction, possibly through an increased NO production, in an experimental model using rat myocytes^(^^24^^)^.

The aim of the present study was to investigate the role of leptin as a possible mediator for the correlation between obesity and cardiovascular dysfunction by investigation of its direct influence upon the functioning human myocardium. We used samples obtained from the right atrium, which usually remains well protected against systematic CVD such as coronary artery diseases and arterial hypertension, and treated them in an *in vitro* model with a supraphysiological dosage of leptin.

## Methods

The study was conducted with permission of the Ethics Board of Rhineland-Palatinate, Germany, and after obtaining individual written consent from the patients for using their disposed tissues, without collecting individual data. No human subjects were involved.

### Obtaining of tissue

We collected in this study tissue samples from the right atrial appendages, which were routinely removed and discarded in the course of cannulation of the right atrium for cardiopulmonary bypass, in patients undergoing cardiac surgery at our hospital. Donors with medical situations that exert a relevant influence on the contractile behaviour of the right atrium were excluded. Potential contra-indications included cardiomyopathy, inflammatory or infective cardiac disease, tricuspid valve incompetence, pulmonary hypertension, severe obesity, age >80 years, preoperative administration of amiodarone, chronic dialysis or similar conditions. We also excluded patients with BMI above 30 kg/m^2^, as those patients have a higher ratio of leptin resistance, which would render the experiments insufficient.

Standard cardiovascular anaesthesia was applied using total intravenous anaesthesia protocols, including propofol and remifentanil. A sufficient circulation was maintained using physiological solutions, noradrenalin and atropine.

### Sample preparation

**Fig. 1.**
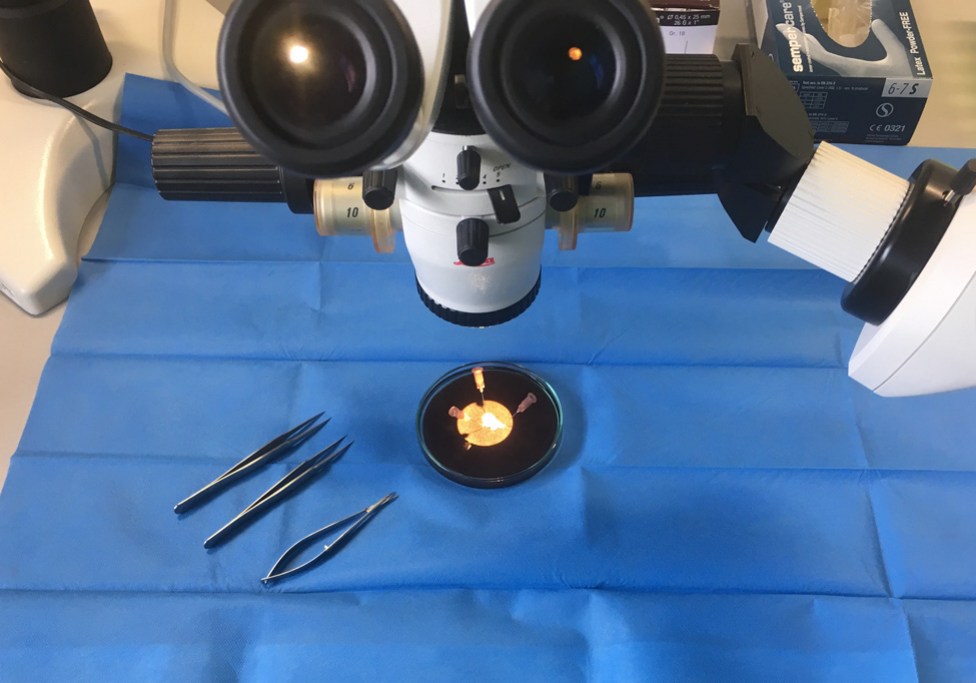
Preparing the samples from the tips of the right atrial appendages.

The tips of the right appendages were removed prior to the cannulation of the right atrium using surgical scissors. Immediately after removal they were transferred to our laboratory in cold (4°C) histidine–tryptophan–ketoglutarate (HTK) solution (prepared by the pharmacy of the University Medical Center of the Johannes Gutenberg University, Mainz, Germany), which contained 15 mm-NaCl, 10 mm-KCl, 4 mm-MgCl.(H_2_O)_6_, 18 mm-histidine.HCl.H_2_O, 180 mm-histidine, 2 mm-tryptophan, 30 mm-mannitol, 0·015 mm-CaCl_2_.(H_2_O)_2_ and 1 mm-ketoglutaric acid. Following this, each sample was manually prepared under the microscope in cold HTK solution to yield muscle specimens measuring 3 × 0·5 × 0·5 mm (see Fig. 1). These muscle specimens were then stored in the dark in cold oxygenated (4°C) HTK solution for 1–48 h. We used this protocol previously in another of our studies^(^^25^^)^.

### Mechanical measurements

**Fig. 2.**
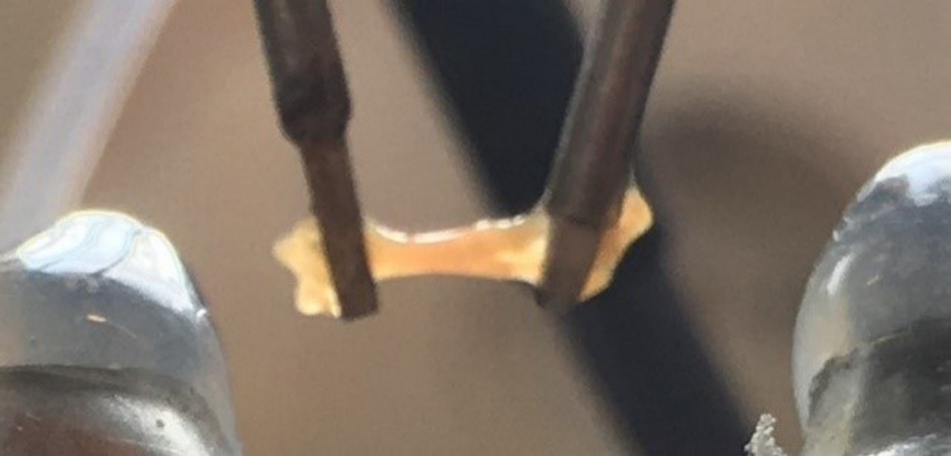
A prepared human atrial sample is fixed between the tweezers of muscle investigation apparatus. This sample measured almost 3 mm long and 0.6 mm wide. It is shown shortly before being immersed in warm Krebs–Henseleit solution and electrically stimulated.

Immediately before the test the tissues were washed with warm Krebs–Henseleit (KH) solution containing (in mm): NaCl, 119·0; NaHCO_3_, 25·0; KCl, 4·6; KH_2_PO_4_, 1·2; MgSO_4_, 1·2; CaCl_2_, 1·3; glucose, 11·0. Thereafter they were mounted horizontally between the tweezers of two identical muscle investigation apparatuses (modified ‘Standard System for Muscle Investigation’; SH Heidelberg) and exposed to a continuous flow of warm (34°C) KH solution steamed with a mix of 95 % O_2_ and 5 % CO_2_ at a rate of 0·5 ml/min each (see Fig. 2).

Following accurate baseline length measuring (slack length), each specimen was stretched to 110 % of its slack length. Then, electrical impulses of 1 ms were applied at a frequency of 75 bpm, using a Stimulator I (Hugo Sachs Elektronik). The voltage was gradually increased until the maximum isometric force of the specimen was reached. Afterwards, the specimens were left to rest for 10 to 30 min to allow them to reach a steady state before we proceeded with the trial.

### Trial design

**Fig. 3.**
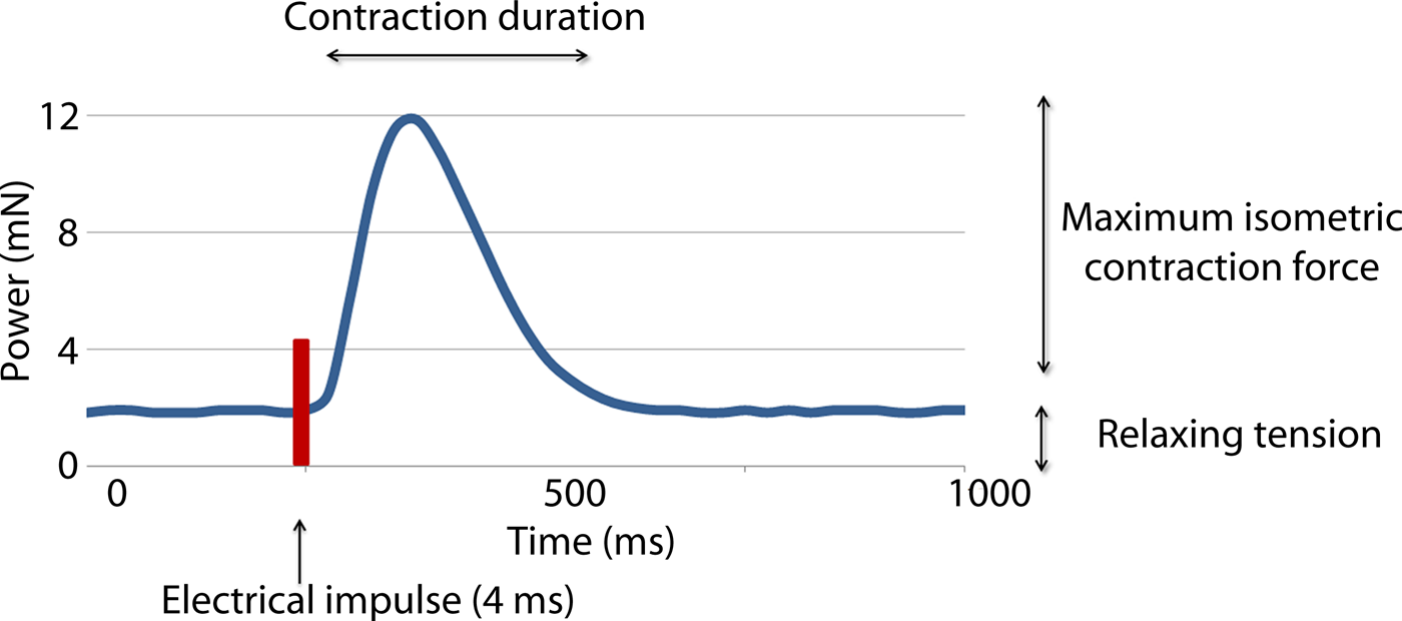
Recording of the forces occurring during one contraction following electrical stimulation of a myocardial sample. Maximum isometric contraction force and the contraction duration were recorded.

Two variables were measured in this study: the active isometric contraction forces (CF) and contraction duration (CD) (see Fig. 3). The measurement was done twice, directly before applying the treatment (CF1 and CD1) and 20 min afterwards (CF2 and CD2). Each measurement lasted 10 min and the average values were recorded in mN.

**Fig. 4.**
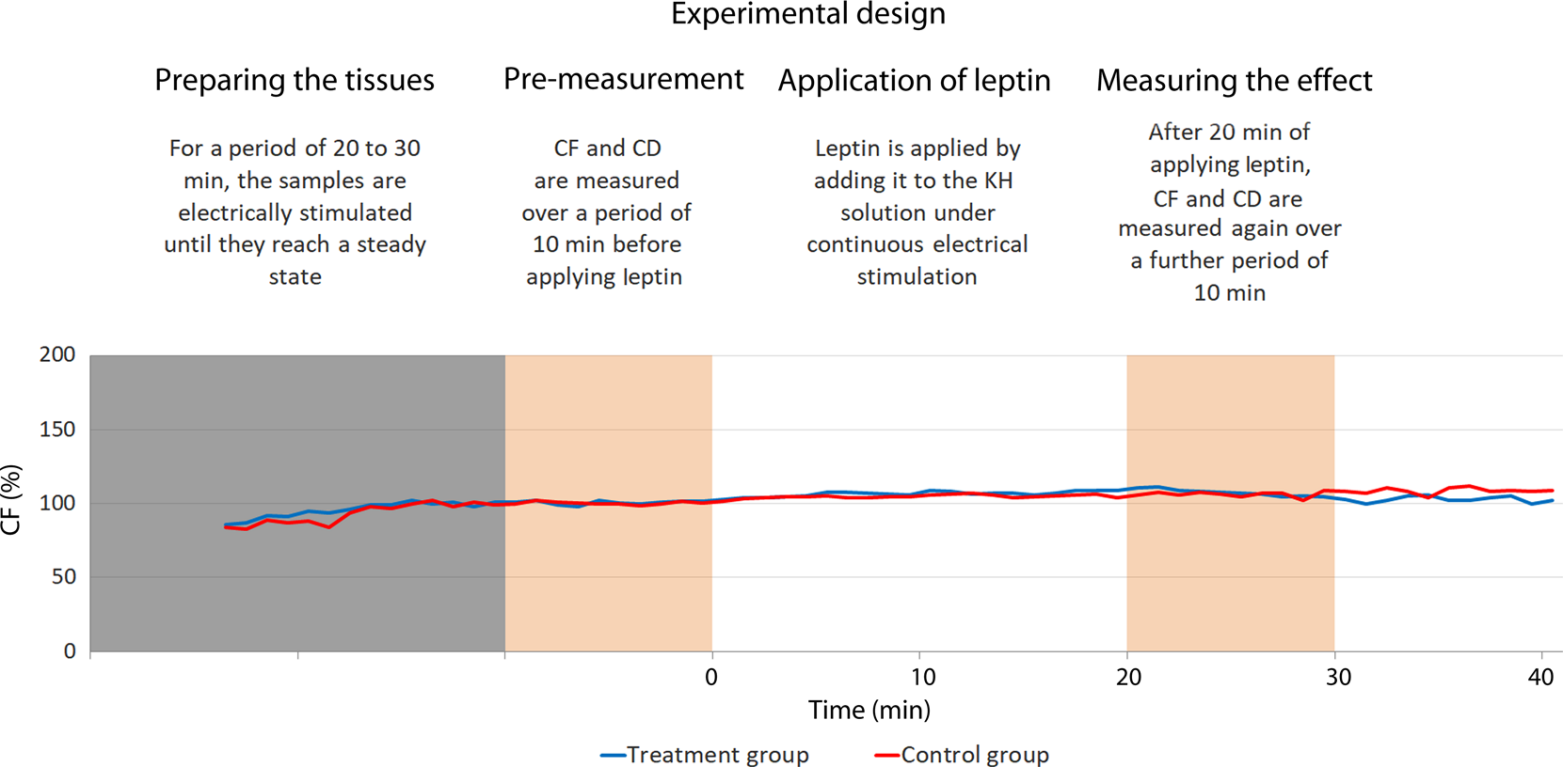
Illustration of the experimental design. The blue line represents the treated sample and the red line represents the control one. Both are harvested from the same patient. In this trial, the treated sample exhibit did not show considerable variation in comparison with the control one. CD, contraction duration; CF, maximal isometric contraction force; KH, Krebs–Henseleit.

Each trial included two samples from the same patient. They were simultaneously electrically stimulated under sustained perfusion to perform isometric contractions. During the stimulation, one sample was tested by treating it with leptin and the other served as a control by treating it with placebo. Fig. 4 illustrates the trial design.

### Leptin application

Human recombinant leptin, expressed in *Escherichia coli*, was obtained as lyophilised powder from Sigma-Aldrich with a purity of >97 %. This was then reconstituted according to the manufacturer's instructions using TRIS-HCl buffer (Sigma-Aldrich) to prepare vials of 1 mg/ml each, which were then stored at 4°C for no more than 3 weeks. On the trial day, leptin was added to the warm Krebs–Henseleit solution by supplanting 100 ml of the KH solution with 0·1 ml of TRIS-HCl buffer containing 100 µg leptin. This yielded a concentration of 1 µg/ml of leptin in KH solution.

The control group was treated with 0·1 ml of TRIS-HCl buffer without leptin as placebo.

### Data acquisition and statistical analysis

We relied on a PicoScope 2204A (Pico Technology), using PicoLog Software for data acquisition. Results were recorded as plain .txt files. The data was then gathered and stored in Excel 2016 (Microsoft Corp.). The CF and CD were calculated using self-developed Excel-VBA-Macros (see Supplementary material).

Statistical analysis was done using IBM-SPSS Statistics (version 23.0.0.0). Categorical variables were described by frequencies and quantitative variables by means and standard deviations.

To lay the foundation for our research, we conducted primary trials using the same model described here to detect the sample size needed in this work. By setting the statistical power to 0·8, ten trials were needed. This decision was also augmented by our experience with this model in previous research^(^^25^^)^.

To adjust for the diversity of the muscle specimens, we divided the values for CF and CD after treatment by the values before the treatment. The results are presented as percentage and the corresponding 95 % CI. Each trial included two samples from the same patient, one in the treatment group and another in the control group. Therefore, we utilised the Wilcoxon signed-rank test to test for differences between the treatment and the control group in the normalised CF and CD data. Two-tailed significance testing was computed as either positive or negative effects of leptin which was to be expected. An *α*-value of 0·05 was chosen for the significance level.

## Results

A total of ten trials obtained from ten different patients were conducted successfully. The mean age of the patients was 67·2 (sd 7·8) years, including four females and six males. Table 1 illustrates their patient profile.

**Table 1.** Summary of the medical profiles and medications of the patients

Medical situations, treatments and medications	Frequency	Rate (%)
Aortic valve replacement	2	20
Mitral valve repair/replacement	1	10
Coronary artery bypass	7	70
Coronary artery disease	8	80
Atrial fibrillation	0	0
Severely reduced ejection fraction	0	0
Moderately reduced ejection fraction	2	20
Diabetes mellitus	6	60
Severe aortic stenosis	2	20
Mitral valve insufficiency	1	10
Arterial hypertension	9	90
Renal insufficiency	0	0
Aspirin	8	80
Clopidogrel	3	30
Thyroxine replacement therapy	2	20
Antihypertensive medications	7	70

Table 2 lists the results of all the trials. We did not observe any variation in the contractile behaviour in the human atrial cardiac tissues after 20 min of treating with high-dosage (1 µg/ml) leptin. CF and the CD remained unchanged in comparison with the control group throughout the experiments. In the treatment group, CF% after 20 min of the treatment with leptin measured 108 (95 % CI 91, 125) % and CD% was 95 (95 % CI 90, 101) %. In the control group, CF% after 20 min of exposure to the placebo measured 105 (90 % CI 84, 126) % and CD was 92 (95 % CI 80, 105) %.

**Table 2.** Results of all trials in absolute values and the value after the treatment divided by the value before treatment (%)

	Treatment group						Control group					
Trial	Contraction force (mN)			Contraction duration (ms)			Contraction force (mN)			Contraction duration (ms)		
no.	Before	After	%	Before	After	%	Before	After	%	Before	After	%
1	1·5	2·3	147	217	179	82	1·1	1·5	140	206	165	80
2	2·8	2·7	97	273	235	86	1·0	1·0	101	268	226	84
3	1·2	1·8	148	253	228	90	3·0	1·6	53	339	223	66
4	5·0	4·3	87	224	239	106	0·7	0·9	121	191	193	101
5	4·7	6·0	126	249	254	102	2·8	2·6	94	267	219	82
6	1·1	1·2	103	224	226	101	1·7	2·1	123	276	259	94
7	1·0	0·9	90	255	249	98	1·9	2·9	150	311	292	94
8	2·1	2·0	91	271	244	90	1·8	1·3	72	211	195	92
9	2·8	2·7	97	224	225	101	1·4	1·4	98	232	311	134
10	2·4	2·2	90	198	190	96	3·1	3·0	98	245	239	98

**Fig. 5.**
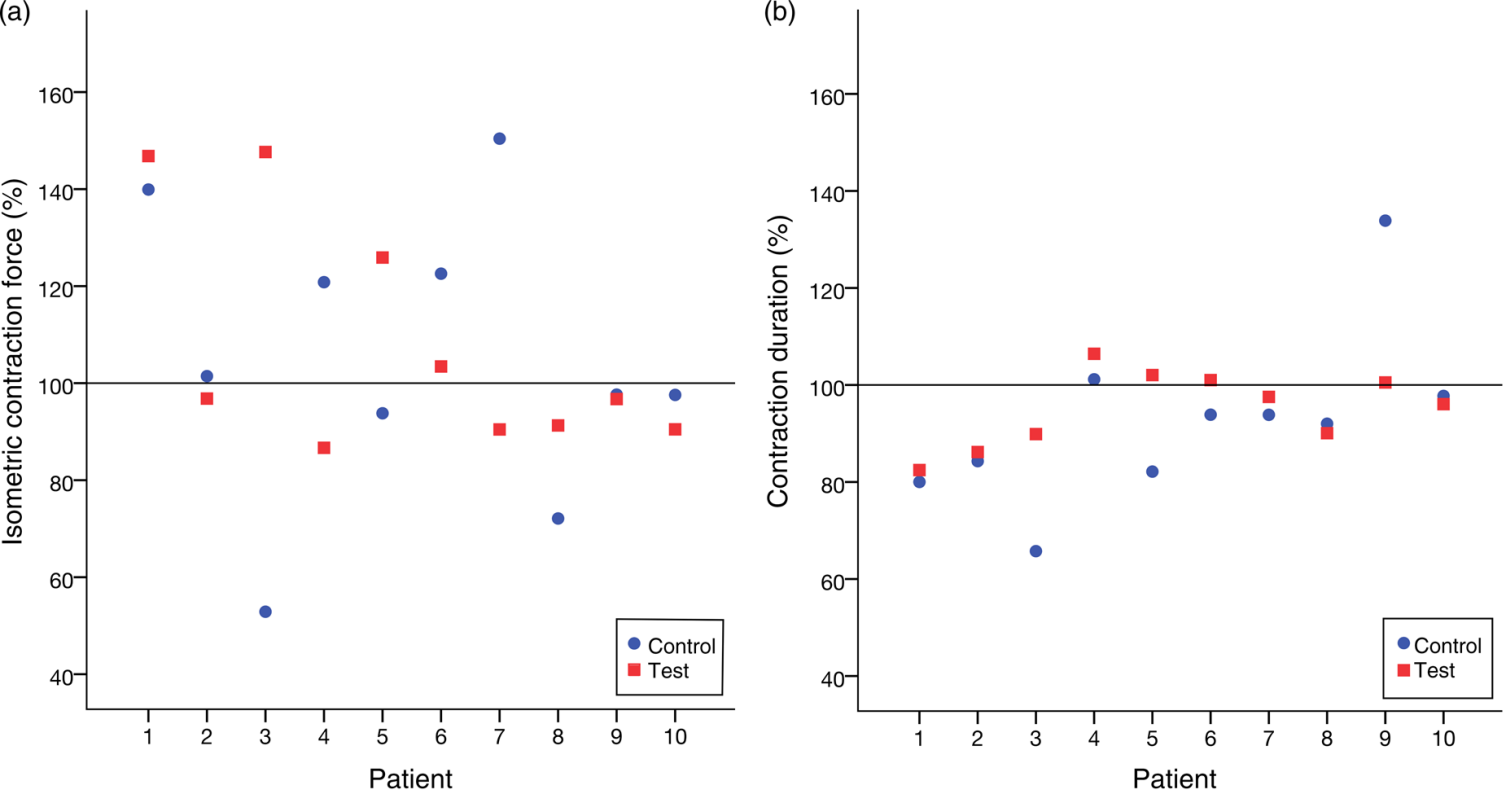
(a) Relative isometric contraction forces of all trials. (b) Relative contraction durations of all trials. No significant variation between the group treated with leptin and the control group was noticed.

The Wilcoxon signed-rank test did not show a significant difference between the treatment and control groups neither for CF (*P* = 0·878) nor for CD (*P* = 0·169). Fig. 5 illustrates this.

## Discussion

Our *in vitro* work shows no effect of high dosage of leptin on the mechanical character of the contraction of human atrial cardiac tissues in our *in vitro* model. To our knowledge, this is the first study to examine the direct effect of leptin upon myocardial contractility, using human tissues. This should help putting together the big picture of how obesity afflicts the cardiovascular system by excluding a direct effect of leptin in this process.

Evidence of leptin production by the heart^(^^26^^)^ and the presence of its receptors there^(^^27^^)^ implicate some regulatory function upon cardiac function. Leptin also influences cardiac metabolism by increasing fatty acid oxidation as shown in isolated working rat hearts^(^^28^^,^^29^^)^. Leptin has been found to increase glucose metabolism as well^(^^30^^)^. *In vitro* administration of leptin to primary cardiac fibroblasts resulted in significant stimulation of pro-collagen Iα, suggesting a role in the remodelling of the cardiac extracellular matrix^(^^31^^)^. Further, it was proven that leptin induced cardiac remodelling associated with hypertrophy^(^^28^^)^. This leptin-associated hypertrophy and a successive diastolic dysfunction were also found in a population-based study (EPIPorto) involving 556 individuals, where higher leptin levels were independently associated with hypertrophy and successive diastolic dysfunction, especially in women^(^^32^^)^. Samuelsson *et al*.^(^^33^^)^ have shown that treating pups from lean Sprague–Dawley rats with a high dosage of leptin led to elevated heart weights and higher night-time (active period) systolic blood pressure. Echocardiography showed an altered left ventricular structure and systolic function in rats treated for 30 d with leptin *v.* the control group and the contractile function was still impaired even 5 months after the treatment with leptin^(^^33^^)^. Larson *et al*.^(^^34^^)^ found that β−3-adrenoreceptors, which usually down-regulate β-adrenergic stimulation, are dependent on leptin and propose this as a possible explanation for the effect of leptin on the myocardium. Further, Breslow *et al*.^(^^35^^)^ found that treating OB/OB mice (mutation in the OB gene which results in the lack of leptin) with leptin restores β−3 adrenoreceptor expression. Lin *et al*.^(^^36^^)^ have proven the presence of leptin receptors on the cell surface of cardiomyocytes obtained from sinus nodes and atrial rat hearts. Further, they found that high-dose leptin reduces heart rate and sometimes causes sinus pauses and ventricular tachycardia. This was present even after treating the tissues with high-dose propranolol, which indicates that this effect is independent of β-adrenoreceptor stimulation. In a meta-analysis of a total of eight case-controlled studies including 1980 patients and 11 567 controls, a significant association of leptin levels with the incidence of CHD and stroke was identified^(^^37^^)^.

Our results do not contradict these studies, as we investigated the direct effect of leptin on the myocardial contractility in the short term, so that negative results do not exclude a potential long-term mechanism. Our setup enabled us also to exclude all other interferences and to isolate the effect of leptin alone, which can also help explaining the lack of effect in our experiments. Besides, leptin is just one possibility for interaction between adipose tissues and the cardiovascular system.

The way that obesity interferes with cardiovascular function is complicated and involves numerous factors, hormonal signalling being just one of them. Another possible explanation is a direct lipid accumulation in the cardiomyocyte and an associated histological damage, leading to cardiomyocyte dysfunction and even apoptosis^(^^38^^)^. There is also the effect of the classical obesity co-morbidity, like arterial hypertension and diabetes mellitus, both of them being considered as risk factors for CVD. Obesity has been found to negatively influence cardiovascular function even in the absence of these co-morbidities^(^^39^^)^.

Finally, the following limitations have to be considered, when comparing our work with other similar studies. The myocardial samples we used were obtained from a typical cardiac surgery population consisting of elderly individuals with a variety of cardiac pathologies. There are known differences between atrial and ventricular myocardium: approximately 15 % smaller atrial cell volume yielding higher surface area:volume ratio; smaller amplitude of systolic Ca^2+^ transients; accelerated rates of decline of systolic Ca^2+^; more sarcoplasmic reticulum (SR)-mediated Ca^2+^ uptake and higher SR Ca^2+^ content^(^^40^^)^. In addition, a higher density of mitochondria is usually found in ventricles^(^^41^^)^. However, regarding the contractile apparatus itself, it was found that the Ca sensitivity of the myofilaments is similar in atrial and ventricular cells^(^^42^^,^^43^^)^. Despite these differences, it seems acceptable to use the atrial tissues in our investigation, especially when the target is merely to identify any possible immediate effect and not to quantify it.

We excluded patients with BMI more than 30 kg/m^2^, as those patients are prone to leptin resistance and therefore a possible leptin effect on the myocardium would be less obvious in this group.

Last, the used concentration of 1 µg/ml (equivalent to 62 nmol/l) is considered supraphysiological as the normal plasma leptin concentration is about 3 to 16 ng/ml and usually less than 100 ng/ml^(^^44^^)^. We chose this concentration to compensate for a range of non-physiological conditions, such as the lack of the carrying proteins in the test medium and the oxidative damage associated with the electrolysis created by the electrical field stimulation. Furthermore, we wanted to discover any possible effect rather than to quantify it, which encouraged us to use this concentration.

### Conclusion

This study shows no effect of high-dosage leptin on the mechanical character of the contractions of human atrial cardiac tissues *in vitro*.

## Supplementary material

For supplementary material accompanying this paper visit https://doi.org/10.1017/jns.2019.6. click here to view supplementary material
